# Rainfall regionalization in Thailand based on statistically validated clustering and its application to spatial rainfall interpolation

**DOI:** 10.1038/s41598-026-48434-1

**Published:** 2026-04-16

**Authors:** Wipawinee Chaiwino, Kuntalee Chaisee, Chalump Oonariya, Ben Wongsaijai

**Affiliations:** 1 Department of Mathematics, Faculty of Science, Chiang Mai University, Chiang Mai, 50200 Thailand; 2 Advanced Research Center for Computational Simulation, Chiang Mai University, Chiang Mai, 50200 Thailand; 3 Office of Research Administration, Chiang Mai University, Chiang Mai, 50200 Thailand; 4 Data Science Research Center, Faculty of Science, Chiang Mai University, Chiang Mai, 50200 Thailand; 5 Department of Statistics, Faculty of Science, Chiang Mai University, Chiang Mai, 50200 Thailand; 6 Climate Center, Thai Meteorological Department, Sukhumvit Rd., Bangkok, 10260 Thailand; 7 Centre of Excellence in Mathematics, MHESI, Bangkok, 10400 Thailand

**Keywords:** Rainfall regionalization, K-means clustering, Principal component analysis, L-moment statistics, Climate sciences, Environmental sciences, Hydrology

## Abstract

Rainfall regionalization plays an essential role in identifying homogeneous rainfall patterns and supporting hydrological and climate analyses. In Thailand, the regionalization adopted by the Thai Meteorological Department (TMD) is primarily based on monsoon wind systems and broad geographic boundaries, which may not adequately represent sub-regional variability in rainfall behavior. This study proposes a data-driven framework to identify homogeneous rainfall regions using monthly rainfall observations from 67 stations across Thailand over the period 1983–2018. Two representations are examined: a standardized representation and a principal component analysis (PCA)-based standardized representation. K-means clustering is applied to both representations, and the resulting clusters are then evaluated using L-moment homogeneity testing, principal component visualization, and statistical and spatial validity indices. The PCA-standardized dataset produces clusters with improved separation and stronger homogeneity relative to the standardized dataset, and the resulting rainfall regions are further interpreted in terms of their physical rainfall characteristics. The practical relevance of the identified regions is further demonstrated through leave-one-out cross-validation comparing inverse distance weighting (IDW), K-nearest-neighbor IDW, and cluster-based IDW approaches using the proposed regions, existing data-driven regions, and TMD regions. The proposed cluster-based IDW approach achieves interpolation error reductions of approximately 9.18–11.55% compared with conventional IDW- and TMD-based alternatives, while providing performance comparable to that obtained using other recent data-driven regional classifications.

## Introduction

Reliable spatial rainfall estimates are fundamental to hydrological modeling and water resource planning^1^. However, their accuracy is frequently compromised by sparse observational networks and insufficient representation of regional rainfall variability^2^. Regionalization offers a solution by identifying areas with homogeneous rainfall characteristics, thereby enabling more accurate spatial analysis^2,3^. This importance is especially evident in monsoon-dominated environments, where strong seasonal forcing, complex topography, and land-sea interactions give rise to substantial spatial variability in rainfall^4,5^. In Thailand, rainfall variability is shaped by strong seasonal circulation together with complex terrain and coastal effects, while rain-gauge coverage is not uniformly distributed across climatologically important areas^6^. Current regionalization by the Thai Meteorological Department (TMD) is based on broad climatic and geographic boundaries^7^. While this classification provides a useful first-order representation of the national climate, it does not ensure homogeneity within regions and may not adequately capture the substantial sub-regional variability observed in rainfall characteristics^8,9^. Observational studies have revealed considerable variability in rainfall characteristics within individual climate regions, potentially limiting the effectiveness of climate monitoring, impact assessments, as well as seasonal prediction^6,8^. This gap between how regions are currently classified and actual rainfall patterns demonstrates the need to redefine Thailand’s rainfall regions using observed climate data instead of depending only on general monsoon patterns^2,10^.

Clustering-based methods have been widely employed to identify homogeneous regions in rainfall and other hydroclimatic variables by grouping stations with similar patterns^3,11^. Various clustering algorithms have been explored for this purpose, including hierarchical clustering, fuzzy C-means, and self-organizing maps^8,12,13^. Among these, K-means clustering, originally proposed by MacQueen^14^, remains one of the most popular methods because of its computational efficiency, ability to handle large station networks, and straightforward centroid-based partitioning^15,16^. This method has been successfully applied to identify homogeneous rainfall regions across diverse climatic settings. In monsoon-dominated areas, it has been used to delineate rainfall zones and analyse their spatial patterns and long-term trends^15^. In other climatic settings, the method has been applied to climate classification in Europe^17^ and has demonstrated good performance in arid and semi-arid areas with limited rain-gauge coverage^18^. More recently, it has been extended to hydrological hazard analysis, including the identification of flood-prone regions through the integration of rainfall and physiographic information^19^. In addition to K-means-based approaches, several studies have explored fuzzy clustering methods, such as fuzzy C-means, which allow stations to belong partially to multiple regions and are particularly useful in data-sparse settings where gradual climatic transitions are expected^20^. Hybrid approaches combining hard K-means and fuzzy C-means have also been applied to analyze rainfall variability and related meteorological characteristics, demonstrating that extensions of centroid-based clustering can be useful when regional boundaries are not sharply defined^21^. However, standard K-means remains a robust and widely adopted baseline for rainfall regionalization^16^ when combined with appropriate preprocessing and validation strategies.

Despite its widespread use, K-means clustering performance in rainfall regionalization is sensitive to how data are prepared and represented^22^. Raw rainfall data are often dominated by differences in magnitude, which can bias clustering toward total rainfall amounts rather than seasonal patterns^23^. To address this issue, many studies employ data standardization and dimensionality reduction techniques, with principal component analysis (PCA) being the most commonly adopted approach^24^. By reducing noise and emphasizing dominant modes of rainfall variability, PCA-based clustering has demonstrated improved regional coherence and has proven effective for precipitation classification at regional scales^25–27^. Its effectiveness has also been demonstrated at the event scale, particularly for distinguishing different precipitation regimes^24,27^. Nevertheless, while PCA is widely adopted, the comparative performance of different preprocessing approaches, in particular simple standardization versus PCA-based transformation, has not been systematically evaluated. It remains unclear whether the added complexity of PCA consistently yields more homogeneous, robust, and practically useful rainfall regions, especially in monsoon-dominated climates.

An important limitation in existing rainfall regionalization studies is the lack of formal homogeneity validation. Many studies interpret clustering results directly without verifying whether the identified regions satisfy statistical homogeneity assumptions^10,28–30^. The L-moment framework developed by Hosking and Wallis^2,31^ addresses this limitation by providing a robust, distribution-free method for assessing regional homogeneity and has been widely adopted in regionalization and regional frequency analysis. Previous studies have demonstrated the effectiveness of L-moment-based homogeneity testing for evaluating rainfall regions derived from clustering in monsoon-influenced and large-scale hydroclimatic settings^3,29^. Its usefulness has also been confirmed in urban-scale analyses and data-sparse environments, where reliable statistical validation is particularly important^22^. More recent methodological developments have introduced network-based clustering frameworks that explicitly enforce homogeneity constraints during the regionalization process^32^. However, in most practical applications, L-moment homogeneity testing is still used primarily as a standalone validation step. This motivates the development of a more integrated validation framework that combines L-moment testing with complementary cluster validity measures for Thailand.

Building on the L-moment framework and addressing the need for integrated and quantitatively validated homogeneity assessment, this study proposes a statistically validated, data-driven framework for identifying homogeneous rainfall regions in Thailand. The main contributions of this study are summarized as follows.

- This study compares two rainfall data representations, a standardized dataset and a PCA-transformed dataset, within a unified K-means clustering framework to assess the influence of data representation on rainfall regionalization in Thailand.
- A statistically validated, data-driven rainfall regionalization framework is developed by integrating L-moment-based homogeneity testing with complementary statistical and spatial cluster validity indices, producing physically meaningful regions that are climatologically consistent with Thailand’s terrain, coastal influences, and dominant seasonal circulation patterns.
- The practical relevance of the identified rainfall regions is evaluated through leave-one-out cross-validation (LOOCV), comparing conventional inverse distance weighting (IDW), K-nearest-neighbor IDW (KNN-IDW), cluster-based IDW using the proposed regions, and cluster-based IDW based on an existing data-driven regionalization^9^ and the operational climatological regionalization of the TMD.

## Methodology

### Methodological workflow

**Figure 1:**
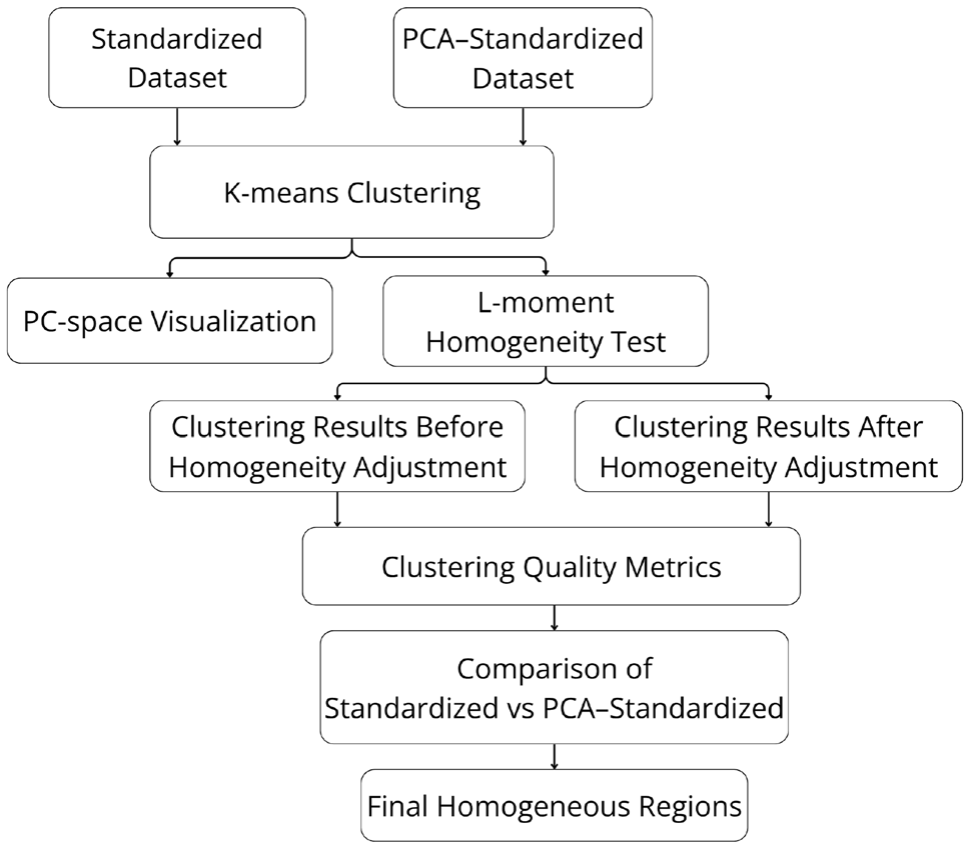
Methodological workflow for rainfall regionalization.

The workflow is organized into three main steps: (1) data processing and clustering, (2) homogeneity assessment, and (3) cluster quality evaluation, as illustrated in Fig. 1. Data processing is first conducted using two rainfall representations: a standardized dataset and a PCA-standardized dataset. K-means clustering is then applied with a fixed number of clusters, and the resulting clusters are visualized in PC space to provide a qualitative assessment of cluster compactness, overlap, and the presence of isolated or boundary stations. Regional homogeneity is formally evaluated using L-moment statistics, where the homogeneity measure is used to identify heterogeneous clusters and discordant stations. When a cluster is classified as heterogeneous, a homogeneity adjustment procedure is applied to remove discordant stations. Clustering results both before and after homogeneity adjustment are retained for subsequent analysis. Cluster quality is further assessed quantitatively using a suite of internal and spatial validity indices to characterize cluster compactness, centroid separation, and spatial regularity. To examine the influence of data representation, clustering results obtained from the two datasets are compared in terms of stability and regional coherence. This comparison leads to the delineation of the final set of homogeneous rainfall regions for Thailand, which are subsequently interpreted in relation to their climatological and hydrological significance.

### Study area and rainfall dataset

**Figure 2:**
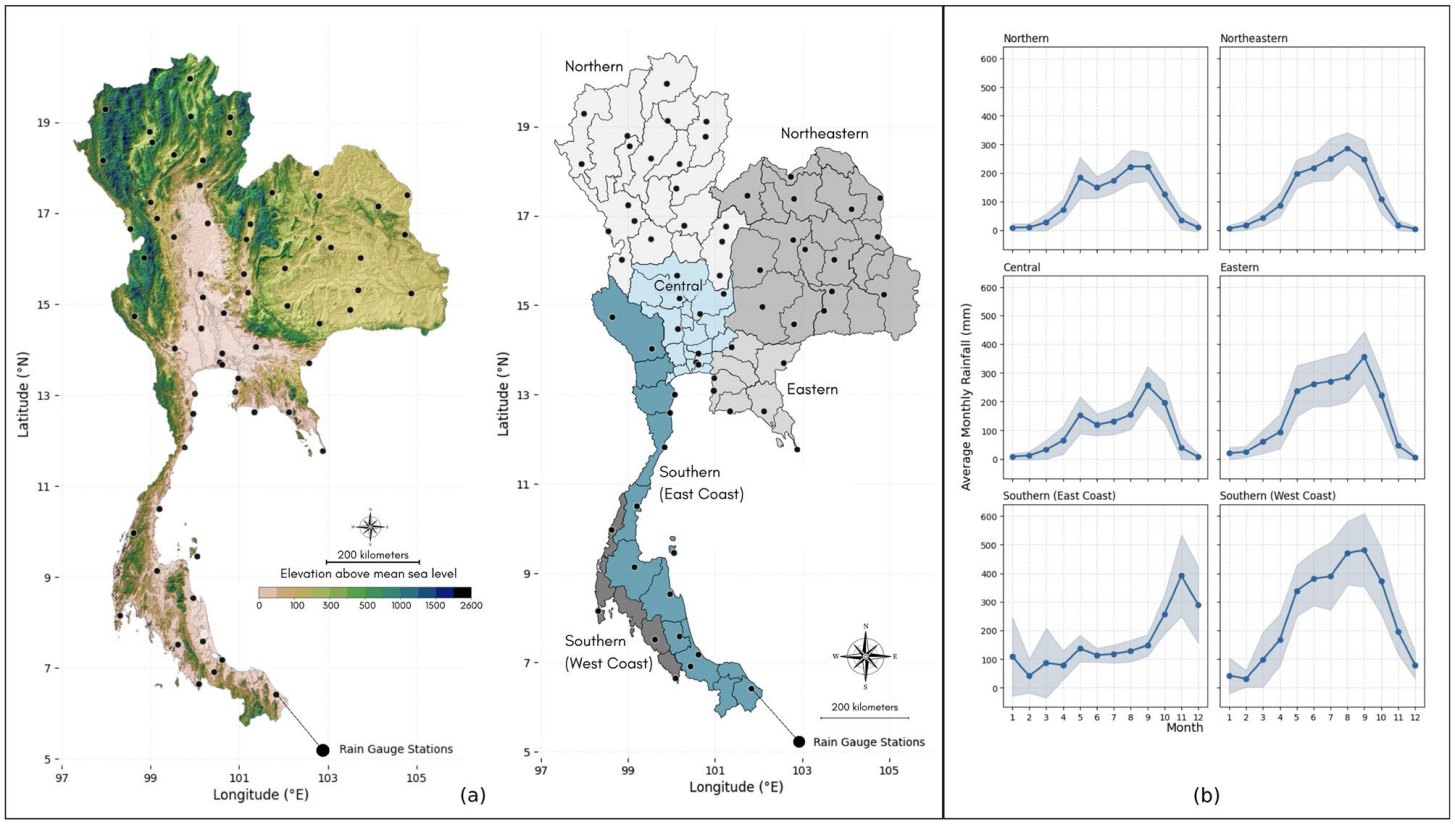
(**a**) Topography of Thailand derived from the HydroSHEDS^33^, including 67 rain-gauge stations. (**b**) Average monthly rainfall regimes across six climatological regions of Thailand.

Thailand is located in mainland Southeast Asia between $5^{\circ }$-$21^{\circ }$N and $97^{\circ }$-$106^{\circ }$E (Fig. 2a), covering an area of approximately 513,000 km$^{2}$. The country comprises 77 provinces and features diverse physiographic units, including mountainous regions in the north and west, the Khorat Plateau in the northeast, the central plain, and coastal zones along the Gulf of Thailand and the Andaman Sea. These physiographic contrasts, together with monsoon circulation, strongly influence the spatial distribution of rainfall. Based on these characteristics, the TMD^7^ classifies Thailand into six climatological regions, namely the northern, northeastern, central, eastern, southern east coast, and southern west coast regions, as illustrated by the different colours in Fig. 2a, which also shows the spatial distribution of the 67 rain-gauge stations used in this study across Thailand.

The monthly rainfall dataset used in this study is obtained from daily observations collected at meteorological stations across Thailand. The dataset spans the period 1983–2018, providing 36 years of continuous monthly rainfall observations. Although data completeness varied across stations, the proportion of missing daily rainfall values remained below 3$\%$. These missing observations were estimated using inverse distance weighting during the data preparation stage. In Fig. 2b, the average monthly rainfall regimes for each climatological region are characterized by solid lines denoting regional means and shaded bands representing $\pm 1$ standard deviation. The northern and northeastern regions experience a clear seasonal rainfall cycle, with a rapid increase beginning in May and the highest rainfall occurring during August–September, reflecting the dominant influence of the southwest monsoon. The Central and Eastern regions show similar monsoon-driven seasonality; however, the Eastern region experiences higher late-season rainfall and greater variability, consistent with coastal influences and enhanced convective activity over the Gulf of Thailand. In contrast, the southern peninsula displays a distinct coastal rainfall regime: the West Coast receives intense and highly variable rainfall during the southwest monsoon, whereas the East Coast exhibits a delayed rainfall peak in November–December associated with the northeast monsoon.

### Data processing methods

#### Principal component analysis (PCA)

Prior to PCA, the rainfall data were standardized using z-score normalization so that each station’s monthly rainfall series has zero mean and unit variance. This transformation reduces the influence of differences in rainfall magnitude among stations and ensures that the analysis focuses on variability patterns rather than absolute rainfall amounts. PCA is then applied to the standardized dataset to extract dominant modes of variability and reduce dimensionality prior to clustering^34,35^. The transformation converts correlated rainfall variables into a set of uncorrelated principal components while retaining most of the total variance in the data. The resulting PCA-transformed dataset provides a noise-reduced representation of rainfall variability. This representation is subsequently used as input for the K-means clustering algorithm. Further mathematical details of PCA are provided in Appendix.

#### K-means clustering

The K-means algorithm assigns each station to one of *k* clusters based on Euclidean distance, with cluster centers updated iteratively until convergence. To ensure robustness, multiple random initializations are performed, and the solution with the lowest within-cluster variance is selected using a fixed random seed. Further formulation details are provided in Appendix. Although K-means assumes approximately spherical clusters, it provides a simple and interpretable framework when applied to standardized or PCA-transformed data. This makes it suitable as a baseline approach for identifying homogeneous rainfall regions.

### L-moment homogeneity assessment

L-moments are linear combinations of probability-weighted moments that describe the location, scale, and shape of a probability distribution. Compared with conventional moments, L-moments are less sensitive to outliers and sampling variability, making them particularly suitable for rainfall data.

Let *X* denote a random variable representing monthly rainfall at a given station, with cumulative distribution function *F*(*x*). The theoretical *r*-th probability-weighted moment is defined as

$$\beta _r = \int _0^1 F^{-1}(p)\,p^r\,dp,$$

where $F^{-1}(p)$ denotes the quantile function of *X*. For a finite sample of size *n* with ordered observations $x_{1:n} \le x_{2:n} \le \dots \le x_{n:n}$, an unbiased estimator of $\beta _r$ is given by

$$\hat{\beta }_r = \frac{1}{n}\sum _{k=r+1}^{n} \frac{(k-1)(k-2)\cdots (k-r)}{(n-1)(n-2)\cdots (n-r)}\,x_{k:n}.$$

The first four L-moments are then obtained as

$$\begin{aligned} L_1&= \beta _0, \\ L_2&= 2\beta _1 - \beta _0, \\ L_3&= 6\beta _2 - 6\beta _1 + \beta _0, \\ L_4&= 20\beta _3 - 30\beta _2 + 12\beta _1 - \beta _0, \end{aligned}$$

corresponding to measures of location ($L_1$), scale ($L_2$), skewness ($L_3$), and kurtosis ($L_4$), respectively. To facilitate comparison across stations with different rainfall magnitudes, the dimensionless L-moment ratios are defined as

$$\tau = \frac{L_2}{L_1}, \qquad \tau _3 = \frac{L_3}{L_2}, \qquad \tau _4 = \frac{L_4}{L_2},$$

where $\tau$ is the L-coefficient of variation, and $\tau _3$ and $\tau _4$ represent L-skewness and L-kurtosis.

Potentially anomalous stations within each cluster are first identified using a discordancy measure based on these L-moment ratios. For station *i*, define the vector

$$\textbf{u}_i = \bigl [\,\tau _i,\; \tau _{3i},\; \tau _{4i}\,\bigr ]^{\textrm{T}},$$

where $\tau _i$, $\tau _{3i}$, and $\tau _{4i}$ denote the at-site L-coefficient of variation, L-skewness, and L-kurtosis, respectively. Let $\bar{\textbf{u}}$ denote the mean vector of $\textbf{u}_i$ over all stations in the cluster, and let $\textbf{S}$ be the sample covariance matrix of $\textbf{u}_i$. The discordancy measure for station *i* is defined as

$$D_i = \frac{1}{3} (\textbf{u}_i - \bar{\textbf{u}})^{\textrm{T}} \textbf{S}^{-1} (\textbf{u}_i - \bar{\textbf{u}}).$$

Stations with $D_i$ values exceeding the critical threshold for the corresponding cluster size are classified as discordant and removed prior to homogeneity reassessment.

After screening for discordant stations, cluster-level homogeneity is evaluated using the L-moment-based statistics $H_1$, $H_2$, and $H_3$. Let $N_k$ denote the number of stations in cluster *k*. For station *i*, let $\tau _i$, $\tau _{3i}$, and $\tau _{4i}$ denote the at-site L-moment ratios, computed from the monthly rainfall record with record length $n_i$. Let $\bar{\tau }$, $\bar{\tau }_3$, and $\bar{\tau }_4$ denote the corresponding weighted mean values within the cluster. The observed dispersion measures are defined as

$$V_1 = \sqrt{ \frac{\sum _{i=1}^{N_k} n_i(\tau _i - \bar{\tau })^2}{\sum _{i=1}^{N_k} n_i} }, \qquad V_2 = \sqrt{ \frac{\sum _{i=1}^{N_k} n_i(\tau _{3i} - \bar{\tau }_3)^2}{\sum _{i=1}^{N_k} n_i} }, \qquad V_3 = \sqrt{ \frac{\sum _{i=1}^{N_k} n_i(\tau _{4i} - \bar{\tau }_4)^2}{\sum _{i=1}^{N_k} n_i} }.$$

Each observed dispersion measure $V_j$ ($j=1,2,3$) is compared with its sampling distribution obtained from $N_{\text {sim}}$ Monte Carlo simulations generated from a homogeneous parent distribution with matching record lengths. Let $\mu _{V_{\text {sim}}}$ and $\sigma _{V_{\text {sim}}}$ denote the mean and standard deviation of the simulated dispersion values, respectively. The homogeneity statistics are then defined as

$$H_1 = \frac{V_1 - \mu _{V_{\text {sim}}}}{\sigma _{V_{\text {sim}}}}, \qquad H_2 = \frac{V_2 - \mu _{V_{\text {sim}}}}{\sqrt{\sigma _{V_{\text {sim}}}^2 + \sigma _{\text {at-site}}^2}}, \qquad H_3 = \frac{V_3 - \mu _{V_{\text {sim}}}}{\sigma _{V_{\text {sim}}}(1 + \lambda _3/6)},$$

where $\sigma _{\text {at-site}}$ accounts for sampling variability in the at-site L-moment ratios, and $\lambda _3$ denotes the L-skewness of the simulated parent distribution. Following common practice, the classification of cluster homogeneity is based primarily on the $H_1$ statistic, while $H_2$ and $H_3$ are reported for completeness and to provide diagnostic insight into differences in distributional shape as reflected by L-skewness and L-kurtosis. A cluster is regarded as acceptably homogeneous when $H_1 < 1.0$, possibly heterogeneous when $1.0 \le H_1 < 2.0$, and definitely heterogeneous when $H_1 \ge 2.0$.

Clusters identified as heterogeneous are subsequently refined through an iterative adjustment procedure that combines geometric proximity and L-moment-based statistical consistency. In this procedure, stations are represented in a reduced feature space, where inter-station similarity is quantified using Euclidean distances between cluster centroids. In the first step, stations exhibiting large discordancy values were reassigned to neighboring clusters with closer centroids, provided that such reassignment reduced within-cluster dispersion and improved L-moment homogeneity. In the second step, if the recalculated $H_1$ statistic of a cluster remained above the homogeneity threshold, the entire cluster is merged with its nearest neighboring cluster based on centroid proximity and similarity in L-moment ratios. This two-step procedure was repeated until all clusters satisfied the homogeneity criterion or no further improvement was achieved. By jointly considering geometric similarity and L-moment-based statistical consistency, the adjustment process ensures that the final rainfall regions are both statistically homogeneous and physically interpretable.

### Clustering validation methods

Cluster quality is evaluated using six complementary validation indices, grouped into statistical and geometric categories. This multi-perspective evaluation is motivated by the fact that a single metric may not sufficiently reflect all aspects of clustering performance, particularly for rainfall data. Statistical indices focus on the compactness and separation of clusters in the feature space and geometric indices assess the spatial coherence and separation of stations based on distance relationships. These indices provide a comprehensive assessment of clustering performance from statistical, spatial, and comparative perspectives. All indices are computed using Euclidean distance to ensure consistency across the analysis and compatibility with the K-means clustering objective function. Similar considerations regarding the role of distance metrics in rainfall regionalization and cluster validation have been discussed in previous studies^36,37^. Further mathematical definitions are provided in Appendix.

#### Statistical validation indices

Statistical indices are used to evaluate clustering performance in the feature space, where stations are grouped based on similarity in rainfall characteristics. Three widely used indices are considered: the Silhouette coefficient (SC), the Calinski–Harabasz index (CH), and the Davies–Bouldin index (DB). These indices assess the balance between intra-cluster compactness and inter-cluster separation, providing an objective measure of clustering quality. The SC evaluates how well each station fits within its assigned cluster compared to other clusters^38^. The CH index measures the ratio of between-cluster separation to within-cluster dispersion^39^. The DB index quantifies the similarity between clusters, where lower values indicate better separation^40^.

#### Geometric validation indices

Geometric indices are used to assess the spatial structure of the clusters by considering distance relationships among stations and cluster centroids. Three measures are employed: intra-cluster distance ($D_{\textrm{intra}}$), inter-centroid distance ($D_{\textrm{inter}}$), and the Hausdorff distance (*H*). The $D_{\textrm{intra}}$ reflects the compactness of clusters by measuring how closely stations are grouped around their centroids. The $D_{\textrm{inter}}$ evaluates the separation between clusters. The *H* captures the maximum discrepancy between cluster boundaries and provides insight into potential overlap between clusters^41^.

## Results

This section presents the results of the rainfall regionalization analysis, focusing on the spatial structure, statistical homogeneity, and overall quality of the identified clusters. The aim is to evaluate whether the derived rainfall regions are both statistically consistent and hydrologically meaningful. The analysis examines the cluster structure in PC space, where the retained PCs explain approximately 90% of the total rainfall variance, assesses regional homogeneity using L-moment statistics, and compares clustering performance before and after homogeneity adjustment using statistical and geometric validity indices. The number of clusters was selected based on a combination of statistical considerations and domain knowledge, with $k=8$ providing a finer spatial resolution than the six-region classification of the TMD while maintaining statistical homogeneity and climatological interpretability.

### Cluster structure in principal component space

**Figure 3:**
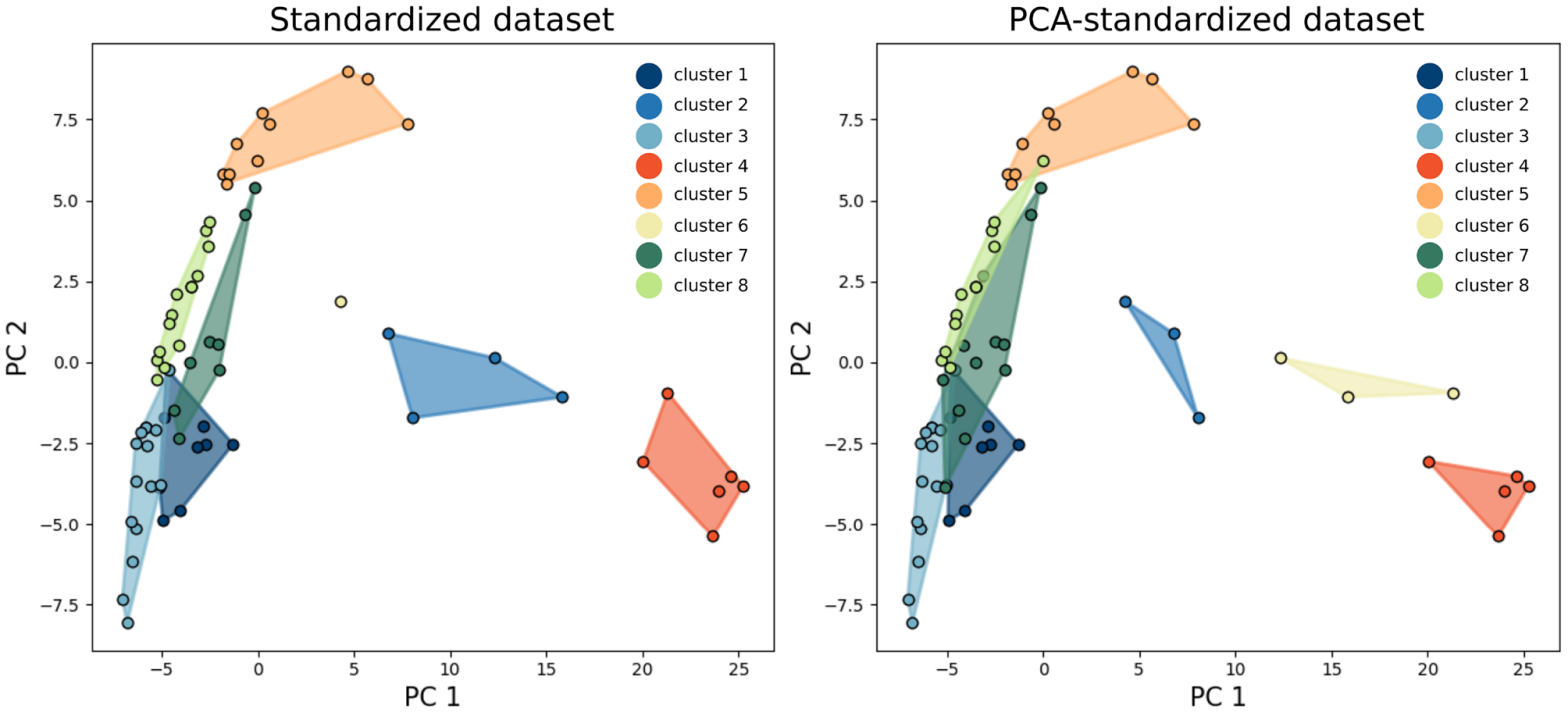
Cluster visualization in PC space for the standardized and PCA-standardized datasets ($k=8$).

**Figure 4:**
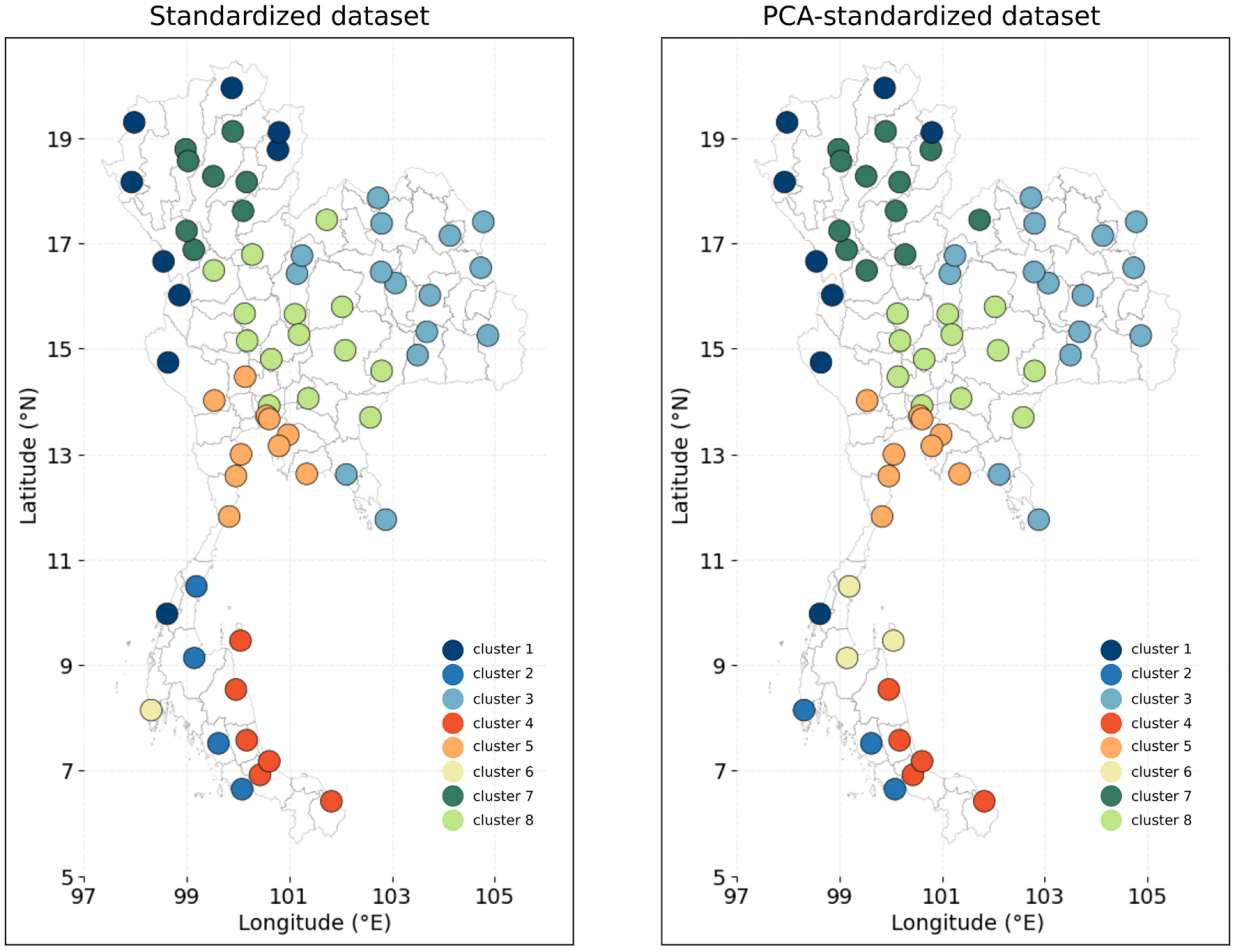
Cluster visualization for the standardized and PCA-standardized datasets ($k=8$).

The cluster structure is first examined in PC space. Figure 3 illustrates the projections of stations onto the first two PCs for both the standardized and PCA-standardized datasets. The two representations exhibit nearly identical configurations in PC space, with stations forming comparable clusters and showing minimal overlap between groups. A notable difference is observed in the standardized dataset, where one cluster consists of a single station appearing isolated in the PC projection. Such singleton clusters have been reported in previous studies as a common outcome of partition-based clustering in the presence of local instability or outlier effects and are generally not considered representative of homogeneous regions^3,42^. Apart from this case, no substantial geometric differences are evident between the two representations. The spatial distributions of cluster memberships are exhibited in Fig. 4. The clustering results derived from the standardized and PCA-standardized datasets are largely consistent at the national scale. In particular, clusters located in the upper part of Thailand (Clusters 1, 3, 5, 7, and 8) exhibit very similar spatial patterns in both results. In contrast, more pronounced differences are observed in southern Thailand (Clusters 2, 4, and 6), where cluster boundaries and spatial extents differ between the two partitions. These discrepancies are mainly confined to the southern region, while the overall large-scale clustering structure remains comparable across both datasets.

### Homogeneity assessment of initial clusters and adjustments

The internal consistency of the initial rainfall regions is evaluated using the L-moment homogeneity measure *H*. According to the commonly adopted criteria, a cluster is classified as homogeneous when $H_1 < 1$, possibly heterogeneous when $1 \le H_1 < 2$, and definitely heterogeneous when $H_1 \ge 2$. During the adjustment process, the identified discordant stations were further examined to determine whether they could be reassigned to other clusters.

Table 1 reports the homogeneity test results for the standardized dataset before and after the homogeneity adjustment. Before adjustment, only Cluster 8 satisfies the homogeneity criterion, while most clusters are classified as definitely heterogeneous and Cluster 3 is identified as possibly heterogeneous. Cluster 6 consists of a single station and therefore cannot be evaluated using the L-moment homogeneity test. After removing discordant stations, all evaluable clusters satisfy the homogeneity criterion, while Cluster 6 remains a singleton and is excluded from homogeneity assessment. These stations were found to be inconsistent with all other clusters and were therefore retained as discordant stations and excluded from the homogeneous regionalization analysis.

Table 2 presents the corresponding results for the PCA-standardized dataset. Prior to adjustment, Clusters 2 and 8 satisfy the homogeneity criterion, whereas the remaining clusters are classified as possibly or definitely heterogeneous. After adjustment, all clusters except Cluster 6 satisfy the homogeneity criterion. Thus, after adjustment, both the standardized and PCA-standardized datasets yield seven homogeneous clusters based on the L-moment criteria. The total number of stations removed during the homogeneity adjustment is identical for both datasets (ten stations). These stations were found to be inconsistent with all other clusters and were therefore retained as discordant stations and excluded from the homogeneous regionalization analysis.

**Table 1:** Homogeneity test results for the standardized dataset.

	Before adjustment					After adjustment				
Cluster	$H_1$	$H_2$	$H_3$	Result	No. of Stations	$H_1$	$H_2$	$H_3$	Result	No. of Stations
1	3.96	5.12	5.00	Definitely heterogeneous	9	-0.69	0.32	1.03	Homogeneous	8
2	3.84	10.74	11.59	Definitely heterogeneous	4	-0.06	3.50	4.11	Homogeneous	3
3	1.35	1.12	-0.07	Possibly heterogeneous	15	-0.42	-0.60	-1.27	Homogeneous	14
4	4.15	7.02	5.94	Definitely heterogeneous	6	0.56	-0.80	-0.64	Homogeneous	5
5	3.54	8.74	16.99	Definitely heterogeneous	10	0.55	2.69	8.96	Homogeneous	7
6	–	–	–	–	1	–	–	–	–	1
7	3.99	4.85	4.49	Definitely heterogeneous	8	0.91	0.82	0.43	Homogeneous	6
8	0.07	0.16	-0.06	Homogeneous	14	0.07	0.16	-0.06	Homogeneous	14

**Table 2:** Homogeneity test results for the PCA-standardized dataset.

	Before adjustment					After adjustment				
Cluster	$H_1$	$H_2$	$H_3$	Result	No. of Stations	$H_1$	$H_2$	$H_3$	Result	No. of Stations
1	4.45	5.51	5.43	Definitely heterogeneous	8	-0.84	-0.22	0.42	Homogeneous	7
2	0.63	0.69	0.45	Homogeneous	3	0.63	0.69	0.45	Homogeneous	3
3	1.35	1.12	-0.07	Possibly heterogeneous	15	-0.42	-0.60	-1.27	Homogeneous	14
4	5.04	8.46	6.76	Definitely heterogeneous	5	0.01	-0.79	-0.59	Homogeneous	4
5	3.19	9.58	19.02	Definitely heterogeneous	9	-0.11	2.81	9.75	Homogeneous	7
6	5.20	12.81	11.30	Definitely heterogeneous	3	5.20	12.81	11.30	Definitely heterogeneous	3
7	3.08	3.63	3.27	Definitely heterogeneous	12	0.21	0.12	-0.26	Homogeneous	10
8	0.98	1.46	1.52	Homogeneous	12	0.98	1.46	1.52	Homogeneous	12

### Cluster validity metrics

Table 3 summarizes the cluster validity and spatial metrics computed before and after homogeneity adjustment for both the standardized and PCA-standardized datasets. Prior to adjustment, the validity indices are broadly comparable between the two data representations and do not clearly favor either approach. After homogeneity adjustment, clearer differences emerge, with the PCA-standardized dataset consistently outperforming the standardized dataset for nearly all metrics. The only exception is the inter-cluster distance metric, $D_{\textrm{inter}}$, for which the values remain comparable. These post-adjustment results indicate that the PCA-standardized representation yields slightly more compact clusters and improved cluster separation.

**Table 3:** Cluster validity and spatial metrics before and after homogeneity adjustment.

	Before adjustment		After adjustment	
Metric	Standardized	PCA-standardized	Standardized	PCA-standardized
SC	0.097	0.093	0.128	0.130
CH	13.464	12.320	12.862	13.030
DB	1.951	1.943	1.730	1.705
$D_{\textrm{intra}}$	12.094	12.016	9.880	9.784
$D_{\textrm{inter}}$	15.346	16.329	15.407	15.324
*H*	21.011	21.484	20.398	19.971
No. of Valid clusters	7	8	7	7

### Homogeneous rainfall regions

The final homogeneous rainfall clusters were obtained by removing discordant stations identified through L-moment homogeneity analysis, improving internal consistency within each cluster. One exception is Cluster 6, for which the stations were retained despite the cluster being statistically heterogeneous. This decision was made because Cluster 6 demonstrates a clear spatial pattern and seasonal rainfall characteristics that align closely with the southern maritime rainfall regime reported by Moron et al.^9^. This consistency validates treating Cluster 6 as a meaningful homogeneous region. Following these adjustments, 60 rain-gauge stations were retained in the final analysis.

**Figure 5:**
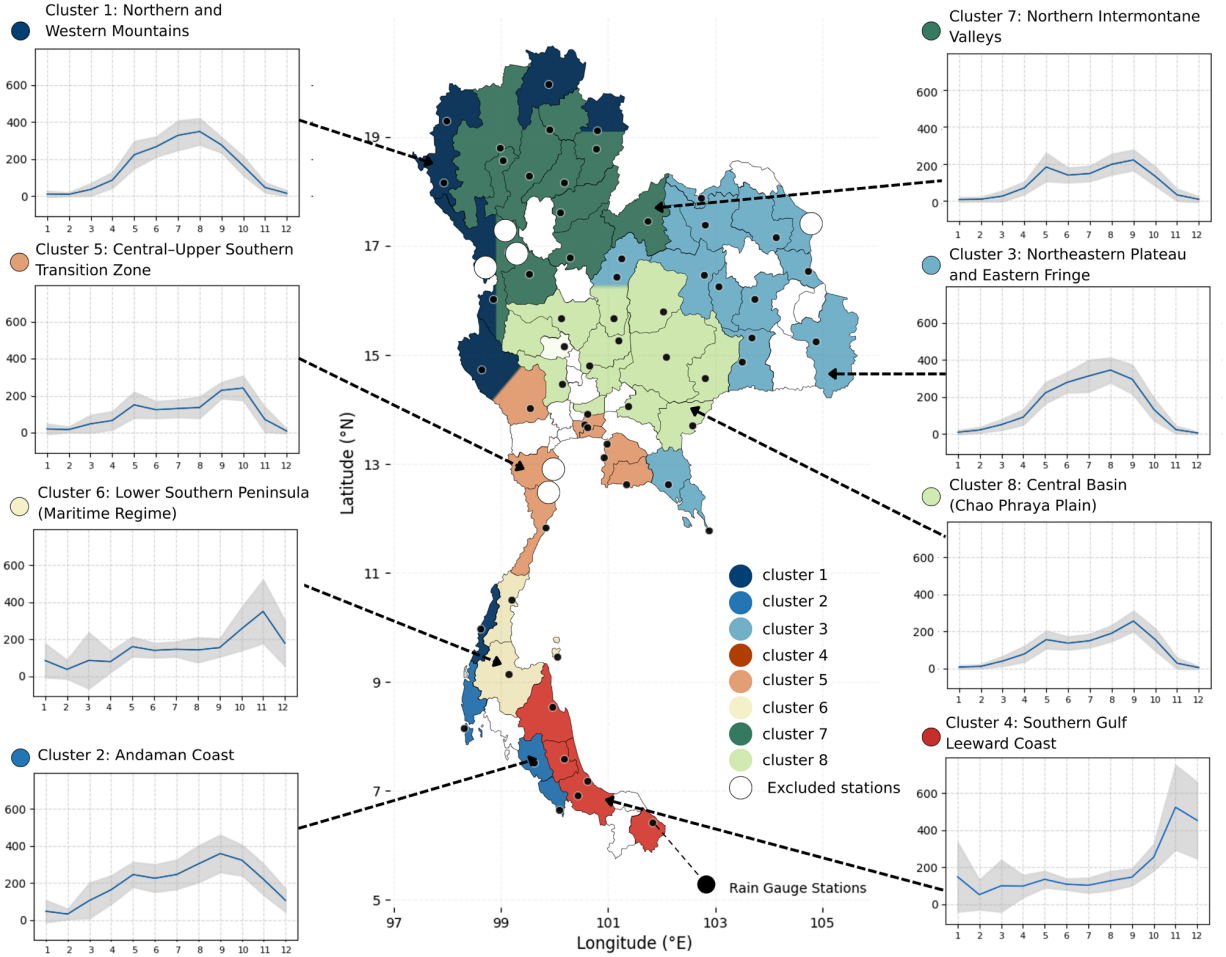
Homogeneous rainfall regions and monthly rainfall patterns from the PCA-standardized dataset.

Table 4 summarizes the distribution of these rain-gauge stations from the TMD climatological regions across the final homogeneous rainfall clusters. The spatial distribution reveals clear regional tendencies. Stations in northern Thailand are mainly grouped into Clusters 1 and 7, while stations from northeastern Thailand are primarily assigned to Cluster 3. Central Thailand is largely represented by Cluster 8, corresponding to the central basin region. Stations from eastern Thailand and the southern peninsula are distributed across several clusters, particularly Clusters 2, 4, 5, and 8, reflecting the diversity of rainfall regimes in coastal and transitional areas. The spatial configuration of the homogeneous rainfall regions is illustrated in Fig. 5, which depicts the full set of rain-gauge stations, including those identified as discordant and removed, with colors indicating cluster membership.

**Table 4:** Distribution of rain-gauge stations across final homogeneous clusters and TMD regions.

Cluster	Dominant Rainfall Regime	TMD Region					
		North	Northeast	Central	East	South	South
1	Northern and Western Mountains	5	–	–	–	1	1
2	Andaman Coast	–	–	–	–	–	3
3	Northeastern Plateau and Eastern Fringe	2	10	–	2	–	–
4	Southern Gulf Leeward Coast	–	–	–	–	4	–
5	Central-Upper Southern Transition Zone	–	–	2	3	2	–
6	Lower Southern Peninsula (Maritime Regime)	–	–	–	–	3	–
7	Northern Intermontane Valleys	9	1	–	–	–	–
8	Central Basin (Chao Phraya Plain)	1	3	6	2	–	–

### Climatological interpretation of homogeneous rainfall regions

From a climatological perspective, the derived regionalization can be interpreted as a compromise between existing classification schemes. In the northern, northeastern, upper-central, and central parts of Thailand, the identified clusters broadly resemble the TMD climatological regions, which are largely controlled by rainfall magnitude and broad-scale geographic factors. In contrast, the partitioning of southern Thailand follows a more monsoon-oriented structure, similar to that reported by Moron et al.^9^, with a clear separation between the lower southern peninsula and the Gulf of Thailand sector.

Accordingly, the rainfall regionalization focuses on the clusters derived from the PCA-standardized dataset. The eight clusters shown in Fig. 5 illustrate distinct spatial rainfall patterns across Thailand, with clear differences between continental and peninsular regions. The spatial distribution and seasonal characteristics of these clusters are broadly consistent with known features of monsoon variability in Thailand^9^. To further illustrate the physical interpretability of the derived regions, a brief characterization of each cluster is provided below. These descriptions highlight how the identified rainfall regimes are shaped by distinct combinations of large-scale and local climate drivers, offering a clearer picture of how the regionalization can be applied in practice alongside its potential for climate interpretation.

**Cluster 1: Northern and Western Mountains** Cluster 1 occupies the windward slopes of the northern and western highlands. Strong southwest monsoon (SWM) flow produces intense orographic rainfall^4,43^, with early-season onset linked to ITCZ migration across Indochina^44,45^. Although the late-June monsoon break temporarily suppresses convection^46,47^, orographic lifting maintains persistent rainfall. A secondary maximum occurs in August–September, reinforced by westward-moving tropical depressions^48,49^. This cluster experiences a long and wet monsoon season.

**Cluster 2: Andaman Coast** Cluster 2 lies directly under SWM inflow from the Andaman Sea and records the highest rainfall totals in Thailand. Early onset is associated with the northward migration of the ITCZ^44,45^, while persistent moisture convergence along the Tenasserim Range generates continuous orographic rainfall^4,43^. The monsoon break is weak because topographic uplift counteracts intraseasonal subsidence^46,50^. Late-season intensification arises from tropical depressions propagating westward from the South China Sea and western North Pacific^48,51^. This cluster represents the most vigorous monsoon regime in Thailand.

**Cluster 3: Northeastern Plateau and Eastern Fringe** Cluster 3 covers the Khorat Plateau and adjacent eastern areas. Rainfall follows a smooth, unimodal monsoon cycle dominated by large-scale circulation. Precipitation increases steadily from April with ITCZ progression^44^ and peaks during July-September under intensified moisture transport from the Bay of Bengal. The monsoon break is relatively weak, yielding a continuous seasonal rise. Late-season rainfall is modulated by monsoon depressions from the South China Sea and western North Pacific^48,52^. Topographic effects are secondary.

**Cluster 4: Southern Gulf Leeward Coast** Cluster 4 lies along the leeward coast of the southern peninsula and is strongly influenced by the northeast monsoon (NEM). Rainfall displays a pronounced bimodal structure. The first peak is associated with ITCZ passage^44,53^, while the dominant October-December peak reflects moisture transport from the Gulf of Thailand under NEM flow^54^. Tropical cyclone remnants further enhance late-season rainfall^48,55^.

**Cluster 5: Central-Upper Southern Transition Zone** Cluster 5 occupies a transition zone between continental and maritime influences. Oscillation of the ITCZ around 10-$13^{\circ }$N produces alternating wet and dry spells during early monsoon months^44^. The monsoon break is evident but short-lived^50,56^. Narrow-peninsula topography enhances upslope rainfall during SWM surges, while NEM flow contributes substantially to late-season rainfall^54^. The resulting regime exhibits mixed continental-maritime characteristics.

**Cluster 6: Lower Southern Peninsula (Maritime Regime)** Cluster 6 lies along the leeward coast of the southern peninsula, where strong northeast monsoon influence produces a clear bimodal rainfall structure. The first peak is associated with ITCZ passage^44,53^, while the dominant October-December peak reflects moisture transport from the Gulf of Thailand under NEM flow^54^. Tropical cyclone remnants further enhance late-season rainfall^48,55^.

**Cluster 7: Northern Intermontane Valleys** Cluster 7 includes intermontane basins such as Chiang Mai, Lampang, and Nan, where valley-mountain circulations modulate convection. Rainfall increases following ITCZ onset^45^ but declines during the monsoon break associated with intraseasonal oscillations^46,57^. A late-season maximum is reinforced by tropical depressions and enhanced western North Pacific monsoon activity^49,52^. Basin geometry produces a distinct, locally modulated seasonal cycle.

**Cluster 8: Central Basin (Chao Phraya Plain)** Cluster 8 represents the central lowlands and the Chao Phraya basin. Rainfall onset coincides with ITCZ migration^44,53^, but the region lies partially in the rain shadow of the Tenasserim Range. The late-June monsoon break produces a noticeable mid-season lull^46,57^. The main rainfall maximum occurs in August–September due to enhanced monsoon depressions^48,58^, forming a classical continental monsoon regime.

## Application: cluster-based rainfall interpolation

In this study, the practical value of the derived homogeneous regions is illustrated through a rainfall interpolation experiment. Specifically, the final stage of the analysis examines whether regionalization improves interpolation accuracy. The LOOCV procedure is employed, in which each station is successively removed and its rainfall value is estimated using the remaining stations. This approach provides an objective and unbiased assessment of interpolation performance. Because the PCA-standardized dataset yields the most consistent and homogeneous rainfall regions, these regions are adopted as the basis for the interpolation experiment.

IDW is a widely used deterministic interpolation technique in geosciences. The classical formulation introduced by Shepard^59^ estimates the rainfall value at an unsampled location $x_0$ as a weighted average of surrounding observations:

$$\hat{z}(x_0) = \frac{\displaystyle \sum _{i=1}^{N} w_i(x_0)\, z(x_i)}{\displaystyle \sum _{i=1}^{N} w_i(x_0)},$$

where $z(x_i)$ is the rainfall observed at station $x_i$, and $w_i(x_0)$ denotes the distance-based weight. The weight is defined as

$$w_i(x_0) = \frac{1}{d(x_0,x_i)^p},$$

where $d(x_0,x_i)$ is the Euclidean distance between locations and $p>0$ is the power parameter controlling the decay of influence with distance. The power parameter is set to $p=2$, which provides a moderate distance decay and is commonly adopted in rainfall interpolation experiments. IDW is widely applied in meteorological and hydrological studies due to its conceptual simplicity and computational efficiency^60^.

A commonly used extension of IDW is the KNN-IDW method, which restricts interpolation to the *k* closest stations to the target location $x_0$^61^. Let $S_n(x_0)$ denote the set of the *n* nearest stations to $x_0$. In the present study, the interpolation uses $n=8$ nearest neighboring stations. The estimator is defined as

$$\hat{z}(x_0) = \frac{\displaystyle \sum _{x_i \in S_n(x_0)} w_i(x_0)\, z(x_i)}{\displaystyle \sum _{x_i \in S_n(x_0)} w_i(x_0)},$$

**Figure 6:**
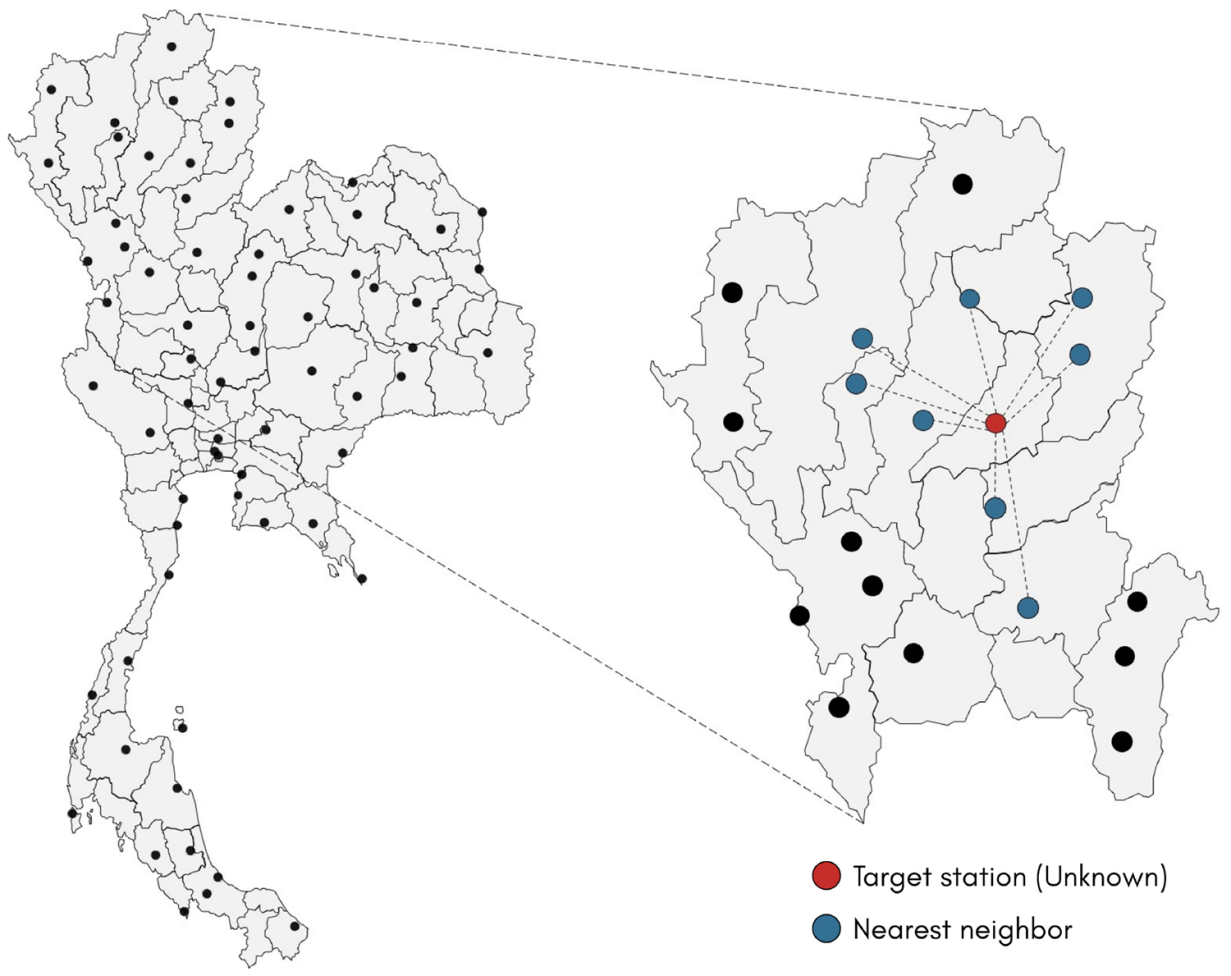
Conceptual diagram of the KNN-IDW interpolation.

Figure 6 illustrates the KNN-IDW concept, where only the nearest neighboring stations are used in the estimation.

To further account for regional rainfall heterogeneity, a cluster-based IDW approach is considered. In this approach, spatial interpolation is performed within each homogeneous rainfall region identified by the clustering procedure, rather than across the entire study domain. For a given target location, only stations belonging to the same rainfall cluster are included in the IDW weighting. This regional constraint is intended to reduce the influence of stations with dissimilar rainfall regimes and thereby improve interpolation accuracy in areas characterized by strong spatial and climatic variability.

Formally, let $\mathcal {C}(x_0)$ denote the homogeneous rainfall cluster to which the target location $x_0$ belongs, as determined by the clustering procedure, and let $S_{\mathcal {C}}(x_0)$ represent the set of stations within the same cluster. The cluster-based IDW estimator is defined as

$$\hat{z}(x_0) = \frac{\displaystyle \sum _{x_i \in S_{\mathcal {C}}(x_0)} w_i(x_0)\, z(x_i)}{\displaystyle \sum _{x_i \in S_{\mathcal {C}}(x_0)} w_i(x_0)},$$

where the weight function $w_i(x_0)$ follows the standard distance-based definition used in classical IDW.

The following analysis presents the LOOCV results comparing the performance of IDW, KNN-IDW, and three variants of cluster-based IDW interpolation. The cluster-based approaches differ in the definition of homogeneous rainfall regions, namely the regions derived in this study, the data-driven regions proposed by Moron et al.^9^, and the operational regional classification of the TMD. For convenience, these approaches are hereafter referred to as cluster-based IDW$_1$, cluster-based IDW$_2$, and cluster-based IDW$_3$, respectively. Table 5 summarizes the overall LOOCV errors for all methods. Among all approaches, cluster-based IDW$_1$ achieves the best overall performance, yielding the lowest MAE and RMSE. Although its error levels are comparable to those of cluster-based IDW$_2$, the proposed approach consistently outperforms conventional IDW and KNN-IDW and clearly improves upon cluster-based IDW$_3$ based on the TMD regions. A similar pattern is observed when errors are examined at the cluster level (Table 6). Cluster-based IDW$_1$ generally produces the lowest errors across most clusters. Exceptions occur for Clusters 2, 4, and 6, where error levels are nearly identical to those of cluster-based IDW$_2$, reflecting the fact that these clusters are identical under both regionalization schemes.

**Table 5:** MAE and RMSE from LOOCV interpolation.

Method	MAE	RMSE
IDW	56.842	108.111
KNN-IDW	55.359	105.164
Cluster-based IDW$_1$	50.274	93.947
Cluster-based IDW$_2$	50.833	94.222
Cluster-based IDW$_3$	57.699	112.530

**Table 6:** MAE and RMSE for each cluster from LOOCV interpolation.

Cluster	IDW		KNN-IDW		Cluster-based IDW$_1$		Cluster-based IDW$_2$		Cluster-based IDW$_3$	
	MAE	RMSE	MAE	RMSE	MAE	RMSE	MAE	RMSE	MAE	RMSE
1	70.762	108.222	73.303	111.843	**66.934**	**100.616**	63.413	97.797	74.324	113.452
2	100.656	137.077	108.683	148.959	**72.101**	**99.301**	**72.101**	**99.301**	107.613	148.292
3	62.384	97.892	57.655	92.732	**55.403**	**88.532**	57.785	95.043	66.056	103.950
4	90.962	143.608	81.907	129.804	**71.005**	**116.349**	**71.005**	**116.349**	78.450	125.899
5	44.476	63.533	39.896	59.012	**39.124**	**58.621**	39.933	62.005	42.455	62.139
6	87.068	118.101	97.221	128.653	**72.363**	**110.375**	**72.363**	**110.375**	91.116	121.227
7	34.046	51.179	33.767	51.666	**33.338**	**51.120**	33.744	52.181	34.257	52.328
8	39.976	57.285	38.857	57.511	**38.439**	**57.001**	39.920	59.598	41.566	61.766

## Discussion

The comparison between the standardized and PCA-standardized datasets shows that the influence of data representation on rainfall regionalization is not immediately apparent. Differences are not clearly visible in the initial PC-space visualizations, and the L-moment analysis yields comparable preliminary cluster structures and a similar number of discordant stations requiring removal for both representations. Clear performance differences emerge only after homogeneity adjustment and quantitative cluster validity assessment, with the PCA-standardized dataset producing clusters that are more compact, better separated, and more spatially coherent.

The homogeneous rainfall regions derived from the PCA-standardized representation correspond broadly to established rainfall regimes in Thailand, likely reflecting the combined influence of large-scale atmospheric circulation and local geographic factors. Since this study relies solely on rainfall data, this correspondence is interpretive rather than confirmatory, but the fact that a purely data-driven approach recovers spatial patterns consistent with known climate boundaries is itself a meaningful result. This suggests that the proposed regionalization framework is capable of capturing meaningful structure in the data that reflects real climatic patterns, rather than producing regions that exist only in a statistical sense. More importantly, regions that are both statistically sound and physically interpretable provide a foundation for practical applications such as regional frequency analysis and design hydrology.

The practical utility of the proposed framework is demonstrated through spatial rainfall interpolation. Incorporating the PCA-derived homogeneous regions into a cluster-based IDW approach reduces cross-validation errors compared with conventional IDW, KNN-IDW, and other cluster-based methods based on the TMD regionalization and the classification of Moron et al.^9^, with the proposed clusters yielding slightly lower interpolation error. These results suggest that cluster-based interpolation can benefit from statistically homogeneous regionalization and that interpolation accuracy is closely related to the quality of the identified clusters.

As with any data-driven study, a number of limitations should be acknowledged when interpreting these findings. The rain-gauge network is relatively limited in density and spatial coverage given Thailand’s diverse terrain and climate, and the uneven station distribution may influence cluster boundary delineation, particularly in data-sparse regions. The observational records may also not fully capture long-term rainfall variability. While K-means was chosen for its practicality and wide use in rainfall regionalization, its assumption of hard cluster boundaries and fixed station assignments may not fully capture the gradual transitions between rainfall regimes across complex terrain. Future research could explore spatially constrained clustering or probabilistic approaches such as Gaussian Mixture Models to better represent transitional patterns and improve spatial continuity across region boundaries. Moreover, further work could also investigate the climatic drivers of the identified rainfall regions by examining how the average rainfall characteristics within each cluster relate to large-scale climate indicators, such as monsoon circulation indices or sea surface temperature anomalies.

## Conclusion

This study developed a statistically validated, data-driven framework for rainfall regionalization in Thailand by integrating K-means clustering, L-moment homogeneity testing, and quantitative cluster validity assessment. The comparison between standardized and PCA-standardized data showed that PCA-based preprocessing improves regionalization performance, yielding homogeneous regions that are statistically robust, spatially coherent, and physically interpretable. The practical value of the framework was further demonstrated through cluster-based spatial rainfall interpolation, which outperformed conventional IDW and other cluster-based approaches. Overall, the results show that data representation and regionalization quality play important roles in interpolation accuracy, and the proposed framework provides a reproducible approach for rainfall regionalization in climatologically diverse regions.

## Supplementary Information

Supplementary Information.

## Data Availability

The data that support the findings of this study are available from the Climate Center, Thai Meteorological Department (TMD) via the TMD Data Service (https://data-service.tmd.go.th). Restrictions apply to the availability of these data, which were used under permission for the current study and are not publicly redistributed. The data are available from the corresponding author upon reasonable request and with permission of the Thai Meteorological Department.

## Declarations

### Code availability

All original programs used for the analysis in this study were written in Python. The authors are willing to share the code and data upon reasonable request. For access to these materials, please contact Ben Wongsaijai at Ben.wongsaijai@gmail.com or Kuntalee Chaisee at kuntalee.chaisee@cmu.ac.th.
