# Supplementary material for: Rainfall regionalization in Thailand based on statistically validated clustering and its application to spatial rainfall interpolation
Source: Sci Rep. 2026 Apr 16;16:17705. doi: 10.1038/s41598-026-48434-1 (PMC13246864; doi:10.1038/s41598-026-48434-1)
Supplement: Supplementary file 1 — Supplementary Information. [file 41598_2026_48434_MOESM1_ESM.zip › Supplementary Material/Supplementary Material.pdf]

Supplementary Information for ”Rainfall regionalization in Thailand based on statistically validated clustering and its application to spatial rainfall interpolation”

A Mathematical Formulation of PCA and K-means

A.1 Principal Component Analysis (PCA)

Let $X \in \mathbb{R}^{n\times d}$ denote the standardized rainfall data matrix, where n is the number of observations (months) and d is the number of stations. The data are mean-centered prior to analysis. The sample covariance matrix $\Sigma \in \mathbb{R}^{d\times d}$ is defined as

$$\Sigma = \frac{1}{n-1} X^{\mathrm{T}} X.$$

The principal components are obtained by solving the eigenvalue problem

$$\Sigma \mathbf{w}_i = \lambda_i \mathbf{w}_i, \qquad i = 1, 2, \ldots, d,$$

where λ_i and $\mathbf{w}_i$ denote the i-th eigenvalue and corresponding eigenvector. The eigenvalues are ordered such that

$$\lambda_1 \geq \lambda_2 \geq \cdots \geq \lambda_d.$$

The projection of the original data onto the principal component space is given by

$$Y = XW_m,$$

where $W_m = [\mathbf{w}_1, \mathbf{w}_2, \ldots, \mathbf{w}_m]$ contains the eigenvectors associated with the m largest eigenvalues, and $m < d$. The proportion of total variance explained by the first m components is defined as

$$\text{Explained Variance Ratio} = \frac{\sum_{i=1}^{m} \lambda_i}{\sum_{i=1}^{d} \lambda_i}.$$

A.2 K-means Clustering

Given a dataset

$$X = \{x_1, x_2, \ldots, x_n\} \subset \mathbb{R}^d,$$

K-means clustering partitions the data into K disjoint clusters $\{C_1, C_2, \ldots, C_K\}$ by minimizing the within-cluster sum of squares:

$$\arg\min_{C_1,\ldots,C_K} \sum_{k=1}^{K} \sum_{x_i \in C_k} \|x_i - \mu_k\|^2,$$

where μ_k is the centroid of cluster C_k, defined as

$$\mu_k = \frac{1}{|C_k|} \sum_{x_i \in C_k} x_i.$$

The algorithm proceeds iteratively through assignment and update steps until convergence.

B Clustering Validation Indices

B.1 Statistical Indices

Silhouette coefficient (SC) For each station i, let a_i denote the mean distance to all other stations in the same cluster, and b_i denote the minimum mean distance to stations in any other cluster. The Silhouette value is defined as

$$S_i = \frac{b_i - a_i}{\max(a_i, b_i)}.$$

Calinski–Harabasz index (CH) The CH index is defined as

$$\mathrm{CH} = \frac{\mathrm{Tr}(B_K)}{\mathrm{Tr}(W_K)} \left(\frac{n - K}{K - 1} \right),$$

where n is the number of stations, K is the number of clusters, and B_K and W_K denote the between-cluster and within-cluster scatter matrices, respectively.

Davies–Bouldin index (DB)

The DB index is given by

$$\mathrm{DB} = \frac{1}{K} \sum_{i=1}^{K} \max_{j \neq i} \left(\frac{\sigma_i + \sigma_j}{d_{ij}} \right),$$

where σ_i is the mean intra-cluster distance of cluster i, and d_{ij} is the Euclidean distance between cluster centroids.

B.2 Geometric Indices

Intra-cluster distance (D_{intra})

$$D_{\text{intra}} = \frac{1}{K} \sum_{k=1}^{K} \frac{1}{|C_k|} \sum_{x_i \in C_k} \|x_i - \mu_k\|.$$

Here, K denotes the number of clusters, C_k is the set of stations assigned to cluster k, $|C_k|$ is the number of stations in that cluster, x_i represents the feature vector of station i, and μ_k is the centroid of cluster k.

Inter-centroid distance (D_{inter})

$$D_{\text{inter}} = \frac{2}{K(K-1)} \sum_{i<j} \|\mu_i - \mu_j\|.$$

Here, μ_i and μ_j denote the centroids of clusters i and j, respectively.

Hausdorff distance (H) For clusters C_i and C_j, it is defined as

$$H(C_i, C_j) = \max \left\{ \sup_{x \in C_i} \inf_{y \in C_j} \|x - y\|, \ \sup_{y \in C_j} \inf_{x \in C_i} \|x - y\| \right\}.$$
